# Comparison of Nanopore and Synthesis-Based Next-Generation Sequencing Platforms for SARS-CoV-2 Variant Monitoring in Wastewater

**DOI:** 10.3390/ijms242417184

**Published:** 2023-12-06

**Authors:** David Garcia-Pedemonte, Albert Carcereny, Josep Gregori, Josep Quer, Damir Garcia-Cehic, Laura Guerrero, Adrià Ceretó-Massagué, Islem Abid, Albert Bosch, Maria Isabel Costafreda, Rosa M. Pintó, Susana Guix

**Affiliations:** 1 Enteric Virus Laboratory, Section of Microbiology, Virology and Biotechnology, Department of Genetics, Microbiology and Statistics, School of Biology, University of Barcelona, 08028 Barcelona, Spain; dgarciapedemonte@ub.edu (D.G.-P.); acarcereny@ub.edu (A.C.); iabid@ksu.edu.sa (I.A.); abosch@ub.edu (A.B.); mcostafreda@ub.edu (M.I.C.); 2 Enteric Virus Laboratory, Institute of Nutrition and Food Safety (INSA), University of Barcelona, 08921 Santa Coloma de Gramenet, Spain; 3 Liver Unit, Liver Diseases—Viral Hepatitis, Vall d’Hebron Institut de Recerca (VHIR), Vall d’Hebron Hospital Campus, 08035 Barcelona, Spain; josep.gregori@gmail.com (J.G.); josep.quer@vhir.org (J.Q.); damir.garcia@vhir.org (D.G.-C.); 4 Centro de Investigación Biomédica en Red de Enfermedades Hepáticas y Digestivas (CIBERehd), Instituto de Salud Carlos III, 28029 Madrid, Spain; 5 Catalan Institute for Water Research (ICRA), 17003 Girona, Spain; lguerrero@icra.cat; 6 Centre for Omic Sciences (COS), Joint Unit Universitat Rovira i Virgili-EURECAT, Unique Scientific and Technical Infrastructures (ICTS), 43204 Reus, Spain; adria.cereto@ce.eurecat.org; 7 Center of Excellence in Biotechnology Research, College of Applied Science, King Saud University, Riyadh 11495, Saudi Arabia

**Keywords:** SARS-CoV-2, Twist RNA, NGS, MiSeq, MinION, Freyja, iVar, LoFreq

## Abstract

Shortly after the beginning of the SARS-CoV-2 pandemic, many countries implemented sewage sentinel systems to monitor the circulation of the virus in the population. A fundamental part of these surveillance programs is the variant tracking through sequencing approaches to monitor and identify new variants or mutations that may be of importance. Two of the main sequencing platforms are Illumina and Oxford Nanopore Technologies. Here, we compare the performance of MiSeq (Illumina) and MinION (Oxford Nanopore Technologies), as well as two different data processing pipelines, to determine the effect they may have on the results. MiSeq showed higher sequencing coverage, lower error rate, and better capacity to detect and accurately estimate variant abundances than MinION R9.4.1 flow cell data. The use of different variant callers (LoFreq and iVar) and approaches to calculate the variant proportions had a remarkable impact on the results generated from wastewater samples. Freyja, coupled with iVar, may be more sensitive and accurate than LoFreq, especially with MinION data, but it comes at the cost of having a higher error rate. The analysis of MinION R10.4.1 flow cell data using Freyja combined with iVar narrows the gap with MiSeq performance in terms of read quality, accuracy, sensitivity, and number of detected mutations. Although MiSeq should still be considered as the standard method for SARS-CoV-2 variant tracking, MinION’s versatility and rapid turnaround time may represent a clear advantage during the ongoing pandemic.

## 1. Introduction

Since the early beginning of the pandemic caused by SARS-CoV-2, efforts have been made to monitor both the circulation and evolution of the virus at the community level. Wastewater-based epidemiology (WBE) has turned out to be a cost-effective early warning tool to monitor the spread of the virus and the upsurge of new variants and their circulation [1]. For this purpose, next-generation sequencing (NGS) information becomes essential, and many countries have implemented wastewater surveillance systems that have become informative tools to support public health decision-making processes regarding the SARS-CoV-2 pandemic [2,3].

Despite sewage being a very complex matrix containing mixtures of variants, NGS may provide reliable qualitative and relative quantitative data. Qualitative data give information of which variants are present in a given sample based on the signature mutations defining each variant, whereas quantitative data show the proportions of these variants. Nevertheless, some studies warn about the reliability of using sequencing results to quantify the abundance of variants in wastewater due to its lower sensitivity in comparison with real-time PCR-based assays [4]. In the context of a pandemic where results are required to take action, fast sequencing techniques as NGS are of critical importance.

Nanopore and synthesis-based sequencing techniques are the most commonly used NGS methods. Nanopores are the basis of Oxford Nanopore Technologies (ONT) sequencing platforms whereas synthesis-based methods are the basis of both Illumina and PacBio sequencing platforms. The Nanopore platform allows real-time sequencing of both short and long reads as well as detection of modified bases (i.e., methylation) in both DNA and RNA [5]. The Illumina sequencing platform can only sequence short reads but provides a very high accuracy. The PacBio sequencing platform, even though not as widely used as Nanopore and Illumina for SARS-CoV-2 variant monitoring, allows long-read sequencing and modified base detection with a similar accuracy as Nanopore [6].

In this work, the main objective was to compare MiSeq (Illumina) with MinION (ONT) NGS approaches to evaluate the pros and cons of each method. With this aim, five synthetic RNAs (Twist bioscience, San Francisco, CA, USA) corresponding to SARS-CoV-2 variants of concern (VOC) Alpha, Beta, Gamma, Delta, and Omicron BA.1 were mixed at different proportions. These mixtures, as well as a sample containing only a synthetic RNA for the Wuhan variant, were sequenced with both approaches with the aim to compare their performance in terms of (i) per position error rate (PPER), (ii) output of reads per position (i.e., read depth), (iii) S gene coverage, (iv) detected mutations, and (iv) accuracy of the estimation of the proportion of each VOC. Two variant callers, LoFreq [7] and iVar (embedded in Freyja) [8,9], were also compared. On the one hand, LoFreq was designed to detect variants at very low frequencies and to be able to distinguish them from sequencing errors. It has been successfully tested in quasi-species analysis [7,10], making it a suitable tool to identify SARS-CoV-2 variants. On the other hand, a recently published bioinformatic tool, Freyja, has been created to determine the abundance of SARS-CoV-2 lineages in mixed samples, specially focusing on wastewater samples [9]. For variant calling, Freyja relies on iVar, which was built for viral amplicon-based sequencing [8]. Additionally, six SARS-CoV-2 naturally contaminated wastewater samples were sequenced to evaluate the performance of these NGS/pipelines in real case scenarios.

## 2. Results

### 2.1. Per Position Error Rate (PPER) Calculation

The quality of the generated reads is a critical issue when making decisions based on sequencing data. By sequencing a Twist synthetic RNA corresponding to the original Wuhan SARS-CoV-2 strain, a comparison of the error rate could be carried out. PPER was defined as the total number of detected substitutions per nucleotide as compared to the expected sequence. Although the occurrence of mutations during the RNA synthesis reaction used to prepare Twist synthetic controls could not be completely ruled out, we considered that any unexpected mutation found in the Wuhan Twist RNA control would be a potential sequencing artifact. In our approach, we considered not only the errors that may occur in the sequencing reactions and the basecalling step but also those that may happen during the aligning and variant calling steps [11,12].

Basecalling quality is generally measured by Phred score. ONT Q-scores may differ from Phred scores although not to a great extent [13]. Tools like Nanoplot [14,15] report Phred scores for ONT data. Prior to the calculation of the PPER, the basecalling quality was assessed for the Wuhan Twist. The mean Phred score values were 36.1 (99.98% basecall accuracy), 13.2 (~95.21% basecall accuracy), 14.4 (~96.37% basecall accuracy), and 22.9 (~99.49% basecall accuracy) for MiSeq, MinION R9.4.1 flow cells, MinION R10.4.1 flow cells singleplex data, and MinION R10.4.1 flow cell duplex data, respectively.

In R10.4.1 duplex data, an important number of substitutions corresponding to the five VOCs were detected in the Wuhan Twist RNA, suggesting errors in the read-pairing process probably due to the high similarity of the amplicons sequenced. For the PPER estimation on the duplex data, we performed two different analyses: (i) including all detected sequencing artifacts and (ii) discarding those mutations that corresponded to VOCs. PPER was evaluated using the output of LoFreq and iVar variant callers. With both variant callers, MinION generated a higher PPER than MiSeq except for duplex data analyzed with LoFreq after removing the detected VOC mutations (Table 1).

With iVar, the differences between the two sequencing platforms were clearly larger, with MinION’s R9.4.1 flow cell having 25 times higher PPER than MiSeq (0.236% vs. 0.009%). R10.4.1 flow cell singleplex data reduced the difference with MiSeq, having only 5 times higher PPER than the Illumina platform (0.050% vs. 0.009%). While PPER was generally lower for MiSeq than MinION, data regarding the total number of sequencing artifacts and the frequency range of these errors greatly varied for each sequencing platform/variant caller combination. Of note, MinION consistently reported a higher frequency range of sequencing artifacts, which could be as high as 71.0% in R9.4.1 flow cell and 48.6% and 33.76% in R10.4.1 singleplex and duplex data, respectively, while sequencing artifacts reported by MiSeq only reached frequencies up to 5.3%. These results indicate a lower sequencing accuracy for MinION as compared to MiSeq, although R10.4.1 singleplex data significantly reduced the number and frequency of sequencing artifacts with both LoFreq and iVar.

In an attempt to reduce the high number of sequencing artifacts detected by iVar in MinION data, a 1% mutation frequency cut-off was included. In the R9.4.1 flow cell, even though the number of sequencing artifacts was still high, we were able to discard 95 of these errors (i.e., a reduction of 28%) whereas R10.4.1 flow cell showed a greater reduction of 53% in singleplex data and over 80% in duplex data. In both MinION’s types of flow cell, the PPER showed a lower decrease than in MiSeq since mutations with frequencies <1% had a very low sequencing depth. In the case of MiSeq data, there was an 84% reduction in the number of sequencing artifacts as well as a three times lower PPER because most of the detected mutations were in frequencies <1%. The cut-off was applied to both Twist mixtures and wastewater samples (see below).

Of note, while iVar reported a higher number of sequencing artifacts in MinION R9.4.1 flow cell than in R10.4.1 singleplex data and MiSeq, an opposite behavior was observed when using LoFreq. LoFreq returned 172, 12, and 10 mutations for MiSeq, MinION R9.4.1, and MinION R10.4.1 singleplex data, respectively, while iVar returned 12, 242, and 36, respectively. Both LoFreq and iVar (without the 1% filter) reported a higher number of sequencing artifacts in R10.4.1 duplex data when compared to other MinION approaches (R9.4.1 and R10.4.1 singleplex data).

### 2.2. Coverage and Depth Comparison

An amplicon-based sub-ARTIC V3 approach targeting only the spike gene was used to amplify 10 different mixtures of Twist synthetic RNA controls corresponding to five different VOCs (Alpha, Beta, Gamma, Delta, and Omicron BA.1), as well as six naturally contaminated wastewater samples, in addition to the synthetic RNA corresponding to SARS-CoV-2 Wuhan. From mixes 1 to 6 (see Section 4.1), synthetic RNA controls corresponding to Omicron BA.1 and Delta variants were mixed at different proportions to emulate what happened with these two variants in a real scenario during the 6th wave in Spain, when the Delta variant was displaced by Omicron BA.1 [16]. From mixes 7 to 10, the five different VOCs were mixed at different proportions to cover multiple possible scenarios.

A total of 14 amplicons (A71 to A84) were obtained for each sample and subsequently sequenced with both MiSeq and MinION platforms (R9.4.1 and R10.4.1 flow cells). The covered percentage of the S gene (coverage) and the number of reads per amplicon (depth) were calculated and compared. The average total number of reads obtained for Twist RNA mixtures were of 5.58 *×* 10^8^ ± 8.61 *×* 10^4^ for MiSeq, 2.35 *×* 10^8^ ± 4.81 *×* 10^4^ for MinION R9.4.1 flow cell, 2.50 *×* 10^8^ ± 3.71 *×* 10^4^ for MinION R10.4.1 flow cell singleplex data, and of 6.18 *×* 10^6^ ± 1.05 *×* 10^3^ for MinION R10.4.1 flow cell duplex data. For wastewater samples, the average total numbers of reads were 3.29 *×* 10^8^ ± 6.83 *×* 10^4^, 1.21 *×* 10^8^ ± 2.55 *×* 10^4^, 6.01 *×* 10^7^ ± 1.39 *×* 10^4^, and 1.14 *×* 10^6^ ± 2.91 *×* 10^2^, respectively.

For the Twist RNA mixtures, MiSeq outputted an average total S gene coverage of 98% (95–100%), MinION R9.4.1 flow cell of 96% (92–100%), MinION R10.4.1 flow cell singleplex of 97% (93–100%), and MinION R10.4.1 flow cell duplex of 91% (55–95%). In the case of wastewater samples, MiSeq data covered an average of 100% of the S gene, MinION R9.4.1 data 89% (51–100%), MinION R10.4.1 singleplex data 77% (46–94%), and MinION R10.4.1 duplex data 56% (34–87%). Only those positions with a number of reads ≥100 were considered for the calculations.

**Figure 1:**
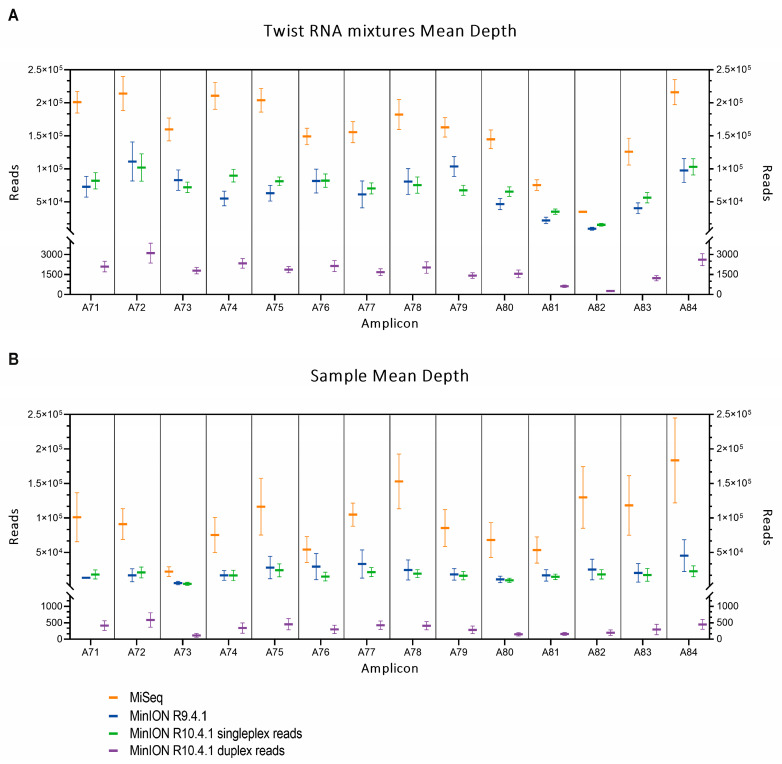
Sequencing depth observed for each amplicon using MiSeq (orange), MinION R9.4.1 flow cell (blue), MinION R10.4.1 flow cell simplex reads (green), and MinION R10.4.1 flow cell duplex reads (purple), for Twist RNA mixtures (**A**) and wastewater samples (**B**). Data represent the average number of reads per amplicon and error bars indicate standard error deviation.

Using the coverage data obtained from the sequencing of the 10 mixtures and the Wuhan Twist synthetic RNA, the mean depth per amplicon was calculated (Figure 1A). Both platforms showed the same trend throughout the spike gene, but MiSeq consistently outputted more reads per amplicon than both MinION flow cells. The lower read depth of the amplicon A82 seen with both sequencing methodologies, which may be due to the overlapping of this amplicon with two Twist fragments.

The same analysis was carried out for the six wastewater samples (Figure 1B). As seen with the Twist mixtures, more reads per amplicon were outputted by the Illumina platform. The almost complete absence of read depth of the amplicon A73 in these samples may be due to the use of ARTIC V3 which was not still optimized for the Omicron variant.

In both Twist RNA mixtures and wastewater samples, the R10.4.1 duplex data showed the lowest number of reads per amplicon.

### 2.3. Comparing the Accuracy of VOC Abundance Estimation

MiSeq and MinION platforms were tested to compare their ability to estimate the abundance of different VOCs present in mixed samples (synthetic RNA mixtures and naturally contaminated samples). The data obtained were analyzed with LoFreq and Freyja and compared to the expected values.

**Figure 2:**
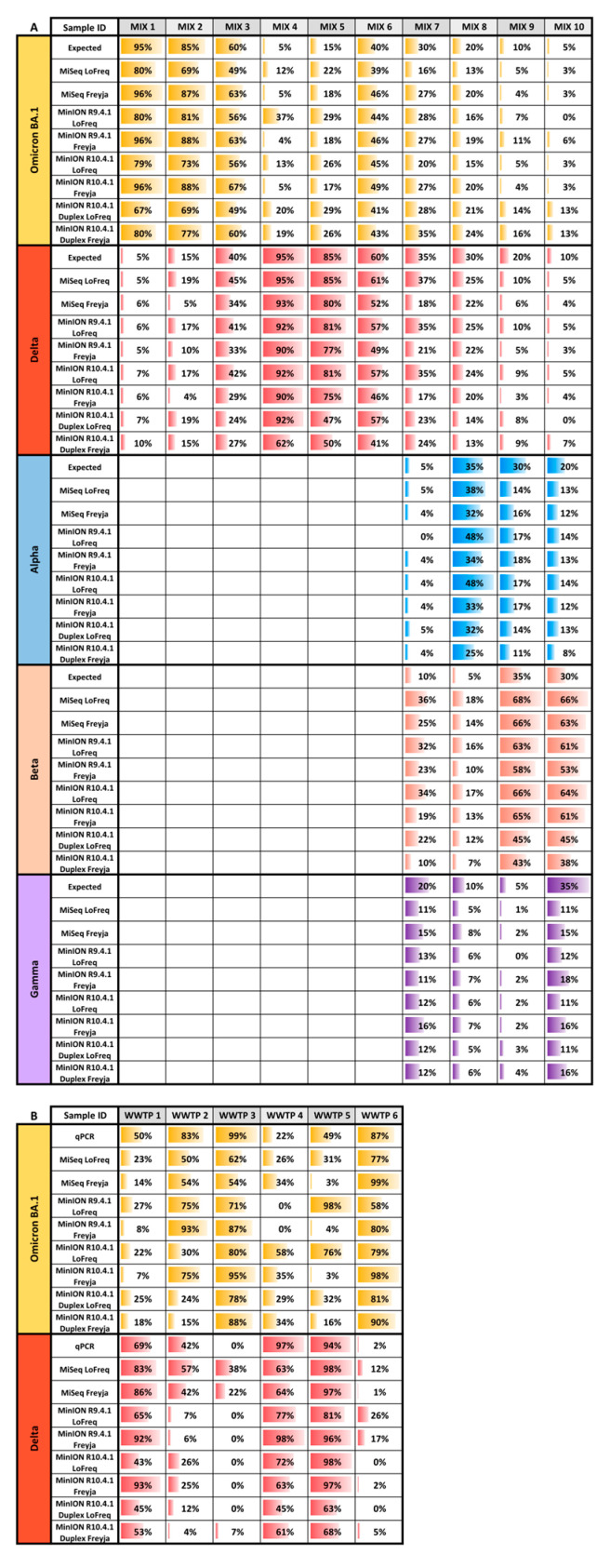
Relative abundance estimations of specific VOCs detected in (**A**) 10 different mixtures of Twist synthetic RNA controls and (**B**) SARS-CoV-2-positive samples collected from 6 different wastewater treatment plants (WWTPs).

Relative VOC abundance estimations measured by the eight different approaches (MiSeq with LoFreq, MinION R9.4.1 with LoFreq, MinION R10.4.1 with LoFreq, MinION R10.4.1 duplex with LoFreq, MiSeq with Freyja, MinION R9.4.1 with Freyja, MinION R10.4.1 with Freyja, and MinION R10.4.1 duplex with Freyja) provided data similar to the expected values (Figure 2A), showing a good accuracy independently of the variant caller used.

Significant positive correlations, determined by the Spearman rank correlation coefficient, between observed and expected results were ascertained for all tested pipelines (Table 2). Of note, with MinION R9.4.1 data, Freyja showed enhanced sensitivity over LoFreq, enabling the detection of all low-frequency variants present at 5%. MinION R10.4.1 flow cell detected all variants present in the mixtures except for duplex data analyzed with LoFreq that missed 5% of Delta VOCs in mix 10. Freyja (iVar) showed a worse correlation than LoFreq with R10.4.1 duplex data due to the detection of non-present VOCs.

Mean deviations between observed and expected percentages were calculated (Table 2). Freyja performed better with MiSeq and MinION R9.4.1 flow cell data and equally to LoFreq with MinION R10.4.1 singleplex and duplex data. Highly biased deviations were obtained for the Beta variant in mixtures 7 to 10 with both sequencing techniques. Consequently, this variant was not considered in the calculations.

In naturally contaminated samples, the percentage of Delta and Omicron BA.1 VOCs was determined by RT-qPCR and compared with the NGS proportion determination. Both sequencing methods were able to successfully detect Delta and BA.1 VOCs present in the samples, even though MinION was not always sensitive enough to detect low-prevalence variants (Figure 2B). In most samples, NGS revealed the presence of only Omicron BA.1 and Delta VOCs, however, in samples 2, 3, and 6 a few signature mutations of Omicron BA.2 were also detected. Consequently, in these samples the common mutations between BA.1 and BA.2 were not included in the abundance estimation. A higher degree of deviation from RT-qPCR values was observed compared to the artificial Twist RNA mixtures, however, significant positive correlations were also observed (Table 2).

Despite the good Spearman rank correlation coefficients of the MinION R10.4.1 duplex data, the five VOCs used in this study were found in all analyzed samples likely due to erroneous read pairing in the duplex basecalling function.

### 2.4. Detected Mutations

An important factor to consider when determining the presence and abundance of a certain variant is the number of detected signature mutations that define this variant. For instance, the recommendation published by the EU in 2021 indicates that at least three genetic markers per variant should be reported for variant identification [17], and Twist RNAs of Alpha, Beta, Gamma, Delta, and Omicron BA.1 VOCs have 4, 3, 7, 4, and 26 signature mutations (SNV), respectively (Figure S1). In the case of the Twist RNA mixtures (Figure S1A), both sequencing platforms gave a similar number of detected mutations when Freyja was used to analyze sequencing data. From 21–29 out of 30 signature mutations were detected in mixes 1–6, which contained only two VOCs, and from 32–36 out of 40 signature mutations were detected in mixes 7–10, which contained five different VOCs. However, with LoFreq fewer signature mutations were detected with MinION R9.4.1 flow cell as compared to MinION R10.4.1 flow cell and MiSeq: only 8–26 out of 30 signature mutations were detected in mixes 1–6 and 11–30 mutations out of 40 expected in mixes 7–10. A similar trend was observed in wastewater samples (Figure S1B), where MinION R9.4.1 flow cell data analyzed with LoFreq consistently detected fewer mutations than Freyja, whereas LoFreq with the R10.4.1 flow cell data was more in line with MiSeq results. Despite the intrinsic variability of these types of samples, MiSeq tended to output more signature mutations than MinION, adding robustness to the results.

MinION R10.4.1 duplex data cannot be directly compared with the other approaches as read-pairing errors are likely to occur, which are a confounding factor when counting the number of detected mutations.

Independently of the sequencing platform, variant caller, and sequencing depth, mutations S371L, S373P, S375F, and K417N of Omicron BA.1 were systematically detected in lower abundances, thus being the first ones to be lost when the concentration of the Omicron BA.1 variant decreased. MiSeq LoFreq was able to detect these mutations in more samples than the other approaches, but with reporting frequencies as low as 0.13% (K417N in mix 4) and, consequently, affecting the variant abundance value.

In Figure S1A,B, those mutations corresponding to VOCs that were not present in the sample are also shown. In wastewater samples from late 2021–beginning 2022, the number of non-present VOCs (Alpha, Beta, and Gamma) that were detected was higher than in Twist RNA mixtures probably due to the lower amount of viral genetic material present in the samples compared to that in the Twist RNA mixtures. This makes the samples more prone to cross-contamination and index hopping (MiSeq) or barcode bleeding (MinION).

## 3. Discussion

NGS technologies are of utmost importance when searching for new variants or mutations that may have an impact on population health. The Illumina platform is generally considered as the gold standard [18,19,20], although ONT is becoming more popular due to its lower cost, turnaround time, and portability [21,22,23], and PacBio also offers SARS-CoV-2-directed resources [24]. Here, we present a comparison between these two platforms, specifically MiSeq (Illumina) and MinION (ONT). Mixtures of known proportions of different Twist synthetic RNAs corresponding to five VOCs were sequenced along with six wastewater samples to assess the differences in terms of error rate, coverage, sequencing depth, estimation accuracy, and sensitivity. Additionally, all these parameters were compared and evaluated using two different bioinformatic pipelines, LoFreq and Freyja, with the aim to determine the effect that different data processing may have on the results.

It is widely believed that MinION does not achieve the MiSeq basecall quality [25,26]. The mean Phred score of Twist RNA mixtures and wastewater samples was 36.0 (99.97% basecall accuracy), 13.0 (~94.99% basecall accuracy), 14.4 (~96.37% basecall accuracy), and 22.9 (~99.49% basecall accuracy) for MiSeq, MinION R9.4.1, and MinION R10.4.1 singleplex and duplex data, respectively. Despite the possible pairing issues that happened with the R10.4.1 duplex read data, there is a remarkable increase in read quality reaching values closer to MiSeq. In our study, we estimated the final PPER of each sequencing platform, i.e., the number of detected errors after variant calling, by sequencing a Twist RNA corresponding to the original SARS-CoV-2 sequence. Our results show that MiSeq has a lower PPER than MinION, independently of the variant caller used, that is in line with previous findings [26]. The largest difference between the two sequencing platforms was found when using iVar with the 1% frequency filter, where MiSeq and MinION R9.4.1 flow cells had a PPER of 0.003% and 0.202%, respectively. Nevertheless, there was a remarkable improvement in MinION’s PPER when using the new R10.4.1 flow cell, having a PPER of 0.036% with LoFreq and of 0.049% with iVar with the 1% cut-off. Contrarily, R10.4.1 duplex data increased the PPER likely due to both read-pairing errors caused by the highly similar amplicons produced when amplifying the five different Twist synthetic RNAs used in the study and the lower sequencing depth of the duplex data.

Two key points to explain the differences found in the PPER between LoFreq and iVar are the number of sequencing artifacts and the frequency of these errors, i.e., the number of reads containing the error. With LoFreq, a high number of sequencing artifacts were reported from MiSeq data, whereas only a few were found in MinION data. With iVar, the opposite behavior was observed particularly with MinION R9.4.1 flow cell and R10.4.1 duplex data. Regarding the frequency of the sequencing artifacts, in MiSeq these were found in low frequencies (up to 5.3%) whereas frequency values went up to 71.0%, 48.6%, and 33.8% in MinION R9.4.1, R10.4.1 singleplex, and R10.4.1 duplex data, respectively. LoFreq was designed under the premise that it is difficult to differentiate between sequencing errors and actual mutations that are present at a very low frequency (<0.05%). To discriminate between sequencing errors and low-frequency mutations, LoFreq estimates the run-specific sequencing error rates based on mapping, base, and alignment qualities [7]. Consequently, on the one hand, MiSeq high read quality enabled LoFreq to report a high number of sequencing artifacts which were mostly found at very low frequencies (<1%), suggesting that some are likely to be Twist synthesis or PCR-induced errors. On the other hand, due to the lower read quality of MinION, LoFreq could only report a few sequencing artifacts which were found at higher frequencies (>5.9% and >1.6% in MinION R9.4.1 and R10.4.1 flow cells, respectively).

Contrarily, iVar (as part of Freyja) does not consider the run-specific sequencing error rate [8]. Our data showed that adding a frequency filter of 1% may help to reduce the background noise, hence, in both sequencing platforms only mutations with frequencies ≥1% are reported. The application of the filter confirmed that in MiSeq most of the detected sequencing artifacts are present at very low frequencies (<1%) whereas in MinION, except for the R10.4.1 duplex data, the frequencies are higher. As expected, MinION R10.4.1 singleplex data fall between MiSeq and R9.4.1 flow cell data, showing a remarkable reduction of the sequencing artifacts when applying the 1% frequency filter, albeit not as high as MiSeq. The differential behavior of these variant callers may explain why, when analyzing MinION data from both Twist mixtures and wastewater samples, iVar outputted more signature mutations than LoFreq, and why with MinION R9.4.1 flow cell data and LoFreq, some variants present at frequencies <5% were lost.

The covered percentage of the gene of interest with enough reads (in our case, >100 reads) is also a parameter of importance when analyzing sequencing data. It has been previously reported that MiSeq provides a better coverage than MinION as well as higher mutation detection [18]. Our results for both Twist RNA mixtures and wastewater samples are in line with these findings. Particularly, when using LoFreq, MiSeq was able to detect more signature mutations than MinION, which, summed to the higher coverage, add robustness to MiSeq results.

With MiSeq data and MinION R10.4.1 flow cell singleplex data, all variants present in the Twist RNA mixtures could be detected even when present at a very low proportion (5%), regardless of the variant caller used. However, with MiniON R9.4.1 flow cell data and LoFreq variant caller, 5% of Alpha, Gamma, and Omicron BA.1 VOCs in mixtures 7, 9, and 10, respectively, could not be detected. MinION R10.4.1 duplex data showed good estimations even though the detection of mutations corresponding to VOCs that were not present in mixtures 1–6 hampers the comparisons with the other approaches. Although not significantly different, correlations between observed and expected VOC frequencies and average deviation values were better when using Freyja than LoFreq for both sequencing methods. The use of the Freyja algorithm has been widely implemented in SARS-CoV-2 wastewater-monitoring studies using Illumina sequencing platforms [9,27,28], but not on ONT sequencing data. To our knowledge, Freyja has only been used with GridION on clinical specimens [29].

In actual wastewater samples, significant positive correlations between measured VOC frequencies and values estimated by duplex RT-qPCR assays were observed with both platforms. MiSeq data performed similarly with both LoFreq and Freyja but showed the weakest correlations (*ρ* = 0.75 and *ρ* = 0.74, respectively) despite outputting more signature mutations than MinION. In contrast, MinION data with both types of flow cells, R9.4.1 and R10.4.1, even though missing some variants present at low frequencies, showed higher correlations with RT-qPCR when Freyja was employed to analyze the sequencing data (*ρ* = 0.81 and *ρ* = 0.82, respectively). It is worth noting that using RT-qPCR abundances may not be as accurate as known Twist RNA concentrations, since many factors (e.g., inhibition) may affect the RT-qPCR results.

While MiSeq typically outperforms MinION based on the studied parameters, it represents a more expensive platform, requiring a fixed space, and both library preparation and run time are generally longer. For instance, the cost of reagents (RT and library preparation) and flow cells required to analyze a single sample in our laboratory, a university-based Spanish research laboratory, was approximately 30% higher for MiSeq as compared to MinION. MinION technology offers an affordable, versatile, portable, and real-time sequencing alternative.

MinION can be of special interest when using wastewater samples as an early warning tool to detect the emergence of new potential variants where sequencing turnaround time is crucial. However, it comes at the cost of losing accuracy and sensitivity when compared to MiSeq [18,30]. It is worth mentioning that the improvement of both flow cell pores and reagent chemistry (R10.4.1 flow cells and kit V14), as well as the improvements in basecalling procedures (duplex reads), narrows the gap with the Illumina platform. Some read-pairing issues seem to happen when generating duplex read data probably due to highly similar amplicons produced when using the ARTIC panel to detect SARS-CoV-2 variants. Duplex data errors may lead to the detection of non-present variants in the samples, suggesting that this approach should be taken with caution when analyzing wastewater samples where a mixture of variants is likely to be present.

It has also been demonstrated that data processing by either LoFreq or Freyja plays a key role in results interpretation, affecting the PPER as well as the accuracy and sensitivity in both sequencing platforms.

## 4. Materials and Methods

### 4.1. Standard RNA Control Mixtures

Solutions of commercially available SARS-CoV-2 synthetic RNA controls (Twist Biosciences, San Francisco, CA, USA) corresponding to Alpha (Control 14, EPI_ISL_710528), Beta (Control 16, EPI_ISL_678597), Gamma (Control 17, EPI_ISL_792683), Delta (Control 23, EPI_ISL_1544014), and Omicron BA.1 (Control 48, EPI_ISL_6841980) were prepared in nuclease-free water, each containing 10^4^ genome copies (gc)/µL. Then, these working solutions were mixed at different proportions as shown in Table 3. Additionally, a solution containing Twist RNA control corresponding to the original Wuhan SARS-CoV-2 sequence (Control 2, MN908947.3) was also prepared at the same concentration. cDNA was synthesized using random hexamers, after a thermal 5 min shock at 65 °C, using SuperScript III enzyme (Thermo Fisher Scientific, Waltham, MA, USA), in the presence of RNaseOUT (Thermo Fisher Scientific, Waltham, MA, USA).

Subsequently, a sub-ARTIC V3 protocol was followed to amplify the spike gene. Briefly, 2 sets of 14 primers corresponding to amplicons 71 to 84 covering the entire S gene were separated into 2 pools (odd and even). Then, 5 µL of the previously obtained cDNA was added to the odd and even pools and a 40-cycle PCR amplification was carried out using the Q5^®^ High-Fidelity DNA Polymerase (New England Biolabs, Ipswich, MA, USA).

### 4.2. Wastewater Samples

Wastewater samples collected from 6 WWTPs located in Spain between 20 December 2021 and 3 January 2022, were chosen for the evaluation of the sequencing methods. With the aim to avoid biases regarding sampling procedures, three 24h composite samples and three grab samples were used. Twenty-four-hour composite samples were collected within the framework of the Catalan Surveillance Network of SARS-CoV-2 in Sewage [31,32,33]. Grab samples were collected within the framework of the VATar COVID-19 Spanish wastewater surveillance project [34,35]. Samples were transported refrigerated (0–4 °C) in a portable icebox, concentrated upon arrival, and analyzed the next day. Viral particles were concentrated from 200 mL of wastewater following an aluminum hydroxide adsorption–precipitation method [36] and RNA extraction was performed using the Maxwell RSC PureFood GMO and Authentication Kit (Promega Corporation, Madison, WI, USA), following the manufacturer’s instructions. The six samples were then confirmed to be positive for SARS-CoV-2 employing both the N1 (CDC) [37] and IP4 (Institut Pasteur) [38] targets.

### 4.3. Library Preparation

Prior to the beginning of the library preparation, PCR products of both standard RNA control mixtures and wastewater samples were purified. The RNA controls were purified with magnetic Kapa Pure Beads (Roche, Basel, Switzerland) and the wastewater samples with the QIAquick Gel Extraction Kit (Qiagen, Hilden, Germany) following the manufacturers’ instructions.

Illumina libraries were prepared using the KAPA HyperPrep Kit (Roche, Basel, Switzerland). Briefly, purified DNA of each sample was (i) quantified using a Qubit^®^ dsDNA HS Assay Kit (Thermo Fisher Scientific, Waltham, MA, USA), (ii) normalized to 1.5 ng/µL with Tris-HCl 10 mM, (iii) end-repaired and A-tailed, (iv) the adaptors were ligated using the Illumina Kapa Single-Indexed Adapter Kit (Set A + B) and purified with Kapa Pure Beads, (v) libraries were amplified by an 8-cycle PCR, purified with Kapa Pure Beads, and quantified with Qubit. Finally, all libraries were pooled and normalized to 4 nM with Tris-HCl 10 mM. MiSeq^®^ Reagent Kit 600v3 cartridges were used to sequence the prepared libraries on the MiSeq^®^ platform (Illumina, San Diego, CA, USA) for 58 h.

MinION libraries to load in the R9.4.1 flow cell were prepared following the Nanopore Classic PCR tiling of SARS-CoV-2 virus protocol [39] with minor changes. Briefly, purified DNA of each sample was (i) quantified using a Qubit^®^ dsDNA HS Assay Kit, (ii) normalized to at least 4 ng/µL with Tris-HCl 10 mM, (iii) end-repaired and A-tailed, (iv) the barcodes were ligated using the EXP-NBD104 and EXP-NBD114 kits, (v) the barcoded samples were pooled and purified with the AMPure XP magnetic beads (Beckman Coulter, Brea, CA, USA), and (vi) the adapters were ligated before the final clean-up with AMPure XP magnetic beads. The obtained library was loaded in a R9.4.1 flow cell (FLO-MIN106D) and sequenced for 72 h using a MinION Mk1C (ONT, Oxford, UK).

At the time of writing, ONT released the new R10.4.1 flow cells and V14 kit chemistry. As stated by ONT, the new R10.4.1 flow cells, in combination with the V14 reagents, enable higher read accuracy (Q20+). Moreover, thanks to a duplex read system in which the complementary strand is read immediately after the template strand and a consensus basecall is performed, qualities ~Q30 or above are claimed to be achieved. We used R9.4.1 and R10.4.1 flow cells and reagents to determine the advantages of these new products when compared to their predecessors and MiSeq.

To prepare the libraries to load into the R10.4.1 flow cell, the ligation sequencing amplicons—Native Barcoding Kit 24 V14 (SQK-NBD114.24) protocol [40] was followed. This protocol was chosen because at the time of writing the Nanopore Classic PCR tiling of SARS-CoV-2 virus protocol used for the R9.4.1 was not yet adapted to the new R10.4.1 flow cells and kit V14 chemistry. However, the steps are very similar to the ones described for R9.4.1 flow cells. The main changes are a clean-up step with beads between the end-repair and the barcode ligation, the use of EDTA instead of heat incubation to stop the barcode ligation, and the addition of BSA to the library prior to flow cell loading. The obtained library was then loaded in an R10.4.1 flow cell (FLO-MIN114). R10.4.1 flow cells have two modes of sequencing, the accurate mode that works at 260 bases per second (bps) and the default mode, at 400 bps. The accurate mode reduces the data yield but favors the quality of the obtained reads, and the default mode prioritizes the data yield at the cost of losing accuracy. The accurate mode was selected for the present study.

Despite the possibility of cleaning and reloading the MinION flow cells, these processes were not carried out in order to compare the performance of MinION with a single library load as was carried out with MiSeq. Had the flow cells been reloaded with additional library material, a higher output would have been expected.

### 4.4. MiSeq Run Settings and Data Processing

Default settings were used to perform the sequencing run.Paired-end FASTQ files were generated from the Illumina MiSeq for each of the synthetic RNA mixtures and sewage samples. The bioinformatic analysis included the next steps: (i) MiSeq R1 and R2 paired ends were used to reconstruct each amplicon using the FLASH v1.2.11 program [41], setting a minimum of 20 overlapping bases and a maximum of 10% mismatches [42], (ii) Phred score was checked using FastQC v0.11.9 and no reads <Q20 were found, (iii) primers were trimmed and low-quality bases (<Q15) were removed from the ends of the sequences using Cutadapt v.3.5 [43], (iv) clean reads were aligned against the region corresponding to the S gene (21563–25384) of the reference genome NC_045512.2 [44] using minimap2 v.2.23 [45]. The resulting BAM file was used for variant calling with both LoFreq v2.1.5 and iVar v1.3.1 (embedded within Freyja v1.3.6) [7,8]. In order to equally process MiSeq and MinION data, single-nucleotide insertions, which are most likely to be sequencing artifacts (mainly present in MinION data), were filtered out and frequencies were adjusted using the BAM alignment positions.

### 4.5. MinION Run Settings and Data Processing

A run length of 72 h with active channel selection activated, a pore scan frequency of 1.5 h, and a minimum read length of 20 bp were set for both R9.4.1 and R10.4.1 flow cells. Real-time basecalling and de-multiplexing were disabled.

For R9.4.1 flow cell data, raw FAST5 files were basecalled and de-multiplexed after the run using Guppy v.6.0.6 into FASTQ files. The basecalling was carried out with the super-accurate (SUP) mode and with a minimum Q-score of 8. The de-multiplexing step was carried out with the options “require_barcodes_both_ends” and “detect_mid_strand_barcodes”.

For R10.4.1 flow cell data, raw FAST5 files were first basecalled with Guppy selecting the specific SUP mode for 260 bps and a minimum Q-score of 8. Using Duplex Tools v0.3.1 [46], the “sequencing_summary.txt” file generated in the basecalling step was processed first by “pairs_from_summary” to identify duplex pair candidates, followed by “filter_pairs” to filter the selected read candidates. After these steps, two files were generated, “pair_ids_filtered.txt” and “pair_ids.txt” which were copied into the folder containing the FAST5 files. Finally, using Guppy, the duplex basecalling was performed over the raw FAST5 files using the 260 bps SUP mode and options “duplex_pairing_mode” and “duplex_pairing_file”.

The resulting FASTQ files were quality checked using Nanoplot v1.40.2 [14] and a mean quality of Q13 was obtained for Twist mixtures and wastewater samples when using the R9.4.1 flow cell. With the R10.4.1 flow cell, a mean quality of Q14.4 was obtained for singleplex data and of 22.9 for duplex data. After QC, reads underwent the same steps described for MiSeq, from step (iii) onwards.

### 4.6. Variant Callers

LoFreq v2.1.5 and iVar v1.3.1 (Freyja v1.3.6) were used to determine the impact of using different variant callers on abundance calculation approaches in the obtained results.

LoFreq was run with “call-parallel” and “no-default-filter” options. Despite using the “no-default-filter” option, alignment qualities, multiple testing correction, and *p*-value threshold filtering on variant quality were still active. This parameter was used with the aim to increase the sensitivity of the variant calling in MinION data.

iVar was run with the “freyja variants” command so default values were used and a variant call format (VCF) file was generated. To calculate the different variant relative abundances, the “freyja demix” command was used and a TSV file was generated with the corresponding variant percentages [9]. Freyja was adapted to work only with the spike gene instead of the whole genome.

### 4.7. Per Position Error Rate (PPER) Calculation

The calculation of the PPER was carried out through the following formula:

PPER = (Nº of reads of the sequencing errors)/(Total S gene reads)(1)

If there are different sequencing errors in the same read, they both add to the PPER value.

### 4.8. VOC Abundance Estimation

Two different approaches were used to calculate the abundance of the present VOCs. When using LoFreq, VOC abundances were calculated as the mean percentage of the signature mutations corresponding to each VOC, including the following criteria: (i) only mutations with a number of reads ≥ 100 were included; (ii) positions with coverage ≥100 where a mutation was expected but not detected were not considered; and (iii) only single-nucleotide variants (SNVs) were used for the calculations. In contrast, when using Freyja (v1.3.6), the relative abundance of the detected variants was automatically calculated and collected in the summarized.tsv file. Of note, as Freyja is based on the UShER [47] phylogenetic tree, deletions are not considered to assign variants.

### 4.9. Relative Quantification of Delta and Omicron BA.1

Two duplex RT-qPCR assays were developed to determine the proportion of Delta- and Omicron BA.1-specific signature mutations: S:Del157/158 (22029_22034DelAGTTCA) and S:Ins214 (22121InsGAGCCAGAA), respectively, as previously performed for other specific mutations [35]. RT-qPCR assays were performed using 400 nM of each primer and 200 nM of each of the two probes targeting genomes with and without the specific signature mutation (Table 4).

RT-qPCR mastermixes were prepared using the PrimeScript One-Step RT-PCR Kit (Takara Bio, San Jose, CA, USA), and the temperature program was 10 min at 50 °C, 3 min at 95 °C, and 45 cycles of 3 s at 95 °C and 30 s at 60 °C. Twist synthetic SARS-CoV-2 RNA controls (Control 2, Control 23, and Control 48) were used to prepare standard curves for genome quantification. The limit of detection (LOD) and limit of quantification (LOQ) were determined for each specific target by running a series of dilutions of the target with 4–10 replicates per dilution. Parameters of all standard curves and estimated LOD and LOQ for the four targets are summarized in Table S1. Percentages of SARS-CoV-2 genomes containing each specific signature mutation were calculated as previously described [35].

## 5. Conclusions

In summary, our study aiming at comparing the performance of MiSeq and MinION sequencing platforms coupled to different bioinformatic pipelines reinforces the idea that Nanopore technology coupled with improved bioinformatic pipelines based on Freyja could serve as a fast tool to generate systematic information on known SARS-CoV-2 variant tracking. However, the Illumina technology should still be considered as the gold standard for the identification and tracking of novel mutations and to confirm the circulation of minority variants.

Nevertheless, the recent improvements made by ONT, despite the need for polishing, offer promising perspectives.

## Figures and Tables

**Table 1:** Accuracy of MiSeq and MinION platforms with LoFreq and iVar using Wuhan SARS-CoV-2 RNA Control.

	MiSeq			MinION											
				R9.4.1			R10.4.1 Singleplex Data			R10.4.1 Duplex Data			R10.4.1 Duplex Data without VOC Mutations		
	LoFreq	iVar	iVar 1% Filter	LoFreq	iVar	iVar 1% Filter	LoFreq	iVar	iVar 1% Filter	LoFreq	iVar	iVar 1% Filter	LoFreq	iVar	iVar 1% Filter
S gene coverage ^1^	100%	100%	100%	93%	93%	93%	93%	93%	93%	87%	87%	87%	87%	87%	87%
Total number of reads ^2^	4.90 × 10^8^	4.90 × 10^8^	4.90 × 10^8^	2.79 × 10^8^	2.79 × 10^8^	2.79 × 10^8^	2.25 × 10^8^	2.25 × 10^8^	2.25 × 10^8^	5.48 × 10^6^	5.48 × 10^6^	5.48 × 10^6^	5.48 × 10^6^	5.48 × 10^6^	5.48 × 10^6^
Number of sequencing artifacts ^3^	172	77	12	12	337	242	10	76	36	31	651	95	2	612	58
Sequencing artifacts frequency range ^4^	0.07–3.5%	0.13–5.3%	1–5.3%	5.9–71.0%	0.02–70.5%	1.1–70.1%	1.6–48.4%	0.03–48.6%	1.2–48.5%	2.17–33.76%	0.21–33.57%	1.00–33.50%	33.63–33.76%	0.21–33.57%	1.00–33.50%
Total sequencing artifacts depth ^5^	1.37 × 10^5^	4.59 × 10^4^	1.57 × 10^4^	1.17 × 10^5^	6.58 × 10^5^	5.65 × 10^5^	8.13 × 10^4^	1.13 × 10^5^	1.10 × 10^5^	7.21 × 10^3^	1.28 × 10^4^	7.24 × 10^3^	9.96 × 10^2^	7.83 × 10^3^	2.92 × 10^3^
Per position error rate (PPER) ^6^	0.028%	0.009%	0.003%	0.042%	0.236%	0.202%	0.036%	0.050%	0.049%	0.132%	0.233%	0.132%	0.018%	0.143%	0.053%

^1^ Percentage of the S gene with a per position depth ≥ 100. ^2^ Sum of all the aligned reads. ^3^ Total number of reported sequencing artifacts passing the filters. ^4^ Minimum and maximum frequencies of the sequencing artifacts. ^5^ Sum of the reads corresponding to the sequencing artifacts. ^6^ Percentage value obtained by dividing the number of reads of the sequencing artifacts by the total number of reads.

**Table 2:** Comparison between observed and expected percentages of the VOCs for Twist mixtures and wastewater samples.

		MiSeq		MinION					
				R9.4.1		R10.4.1 Singleplex		R10.4.1 Duplex	
		LoFreq	Freyja	LoFreq	Freyja	LoFreq	Freyja	LoFreq	Freyja
Twist mixtures	Spearman rank correlation	0.962	0.978	0.938	0.978	0.960	0.969	0.912	0.812
	*p*-value	*p* < 0.05	*p* < 0.05	*p* < 0.05	*p* < 0.05	*p* < 0.05	*p* < 0.05	*p* < 0.05	*p* < 0.05
	Mean deviation	6.6 ± 5.9%	5.8 ± 5.2%	7.1 ± 7.1%	5.6 ± 4.7%	6.7 ± 5.4%	6.8 ± 5.6%	10.0 ± 9.0%	10.0 ± 9.0%
Wastewater samples	Spearman rank correlation	0.747	0.739	0.765	0.813	0.752	0.818	0.834	0.790
	*p*-value	*p* < 0.05	*p* < 0.05	*p* < 0.05	*p* < 0.05	*p* < 0.05	*p* < 0.05	*p* < 0.05	*p* < 0.05
	Mean deviation	20.3 ± 13%	21.3 ± 16%	21.3 ± 14%	17.9 ± 16%	20.2 ± 15%	16.9 ± 16%	23.0 ± 19%	24.0 ± 19%

**Table 3:** Proportion of synthetic RNA controls included in each 10 RNA mixtures.

Sample ID	Omicron BA.1	Delta	Alpha	Beta	Gamma
Mix 1	95%	5%	-	-	-
Mix 2	85%	15%	-	-	-
Mix 3	60%	40%	-	-	-
Mix 4	5%	95%	-	-	-
Mix 5	15%	85%	-	-	-
Mix 6	40%	60%	-	-	-
Mix 7	30%	35%	5%	10%	20%
Mix 8	20%	30%	35%	5%	10%
Mix 9	10%	20%	30%	35%	5%
Mix 10	5%	10%	20%	30%	35%

**Table 4:** Primers and probes used for Omicron BA.1 and Delta duplex RT-qPCR assays.

Mutation	Name	Sequence (5′–3′) ^1^	Length (nt)	Amplicon (pb)
S:Del157/158	For-S21994	TTACCACAAAAACAACAAAAGTTGG	25	83–89
	Rev-S22057	GCTGAGAGACATATTCAAAAGTGCAA	26	
	Probe-Delta	FAM/TGGAAA+G+T+GGAGTTTATT+C+TAGTG/IABkFQ	24	
	Probe-NoDelta	HEX/AGT+GAGTTCA+G+AGTTTATT+CTA+GTG/IABkFQ	25	
S:Ins214	For-S22102	AGGAAAACAGGGTAATTTCAAAAATC	26	140–149
	Rev-S22220	CCAATGGTTCTAAAGCCGAAAA	22	
	Probe-BA.1	FAM/TGCGTGAGC/ZEN/CAGAAGATCTCCCTCA/IABkFQ	30	
	Probe-NoBA.1	HEX/CGCCTATTA/ZEN/ATTTAGTGCGTGATCTCCCTCA/IABkFQ	30	

^1^ +A, +G, +C, +T indicate locked nucleic acids (LNAs).

## Data Availability

The data set supporting the results of this article is available in the Zenodo repository, https://doi.org/10.5281/zenodo.7786559 accessed on 17 April 2023.

## Supplementary Materials

The following supporting information can be downloaded at: https://www.mdpi.com/article/10.3390/ijms242417184/s1.
