# Supplementary material for: Comparison of Nanopore and Synthesis-Based Next-Generation Sequencing Platforms for SARS-CoV-2 Variant Monitoring in Wastewater
Source: Int J Mol Sci. 2023 Dec 6;24(24):17184. doi: 10.3390/ijms242417184 (PMC10743471; doi:10.3390/ijms242417184)
Supplement: Supplementary file 1 [file ijms-24-17184-s001.zip › ijms-2727570-supplementary.pdf]

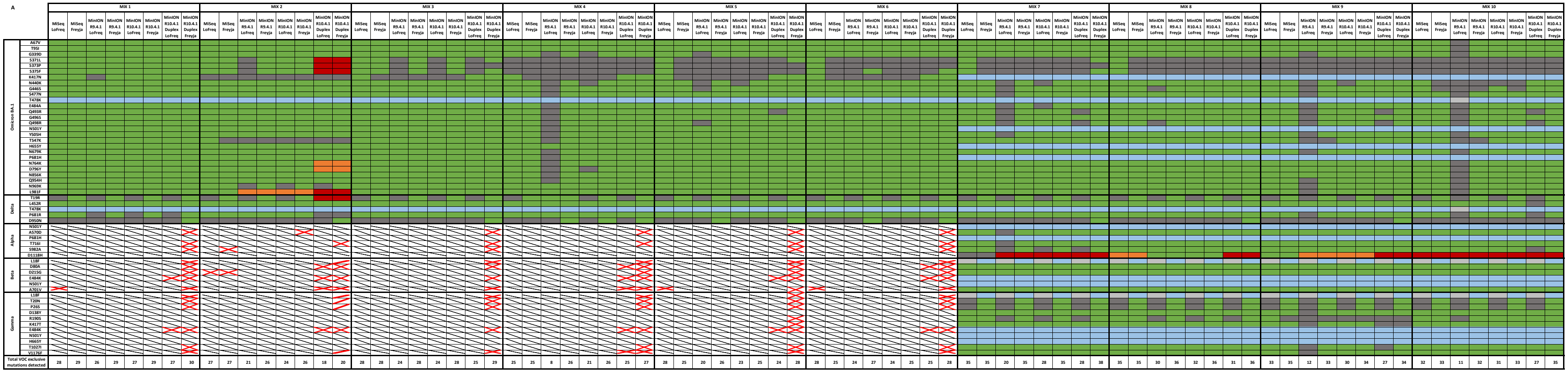

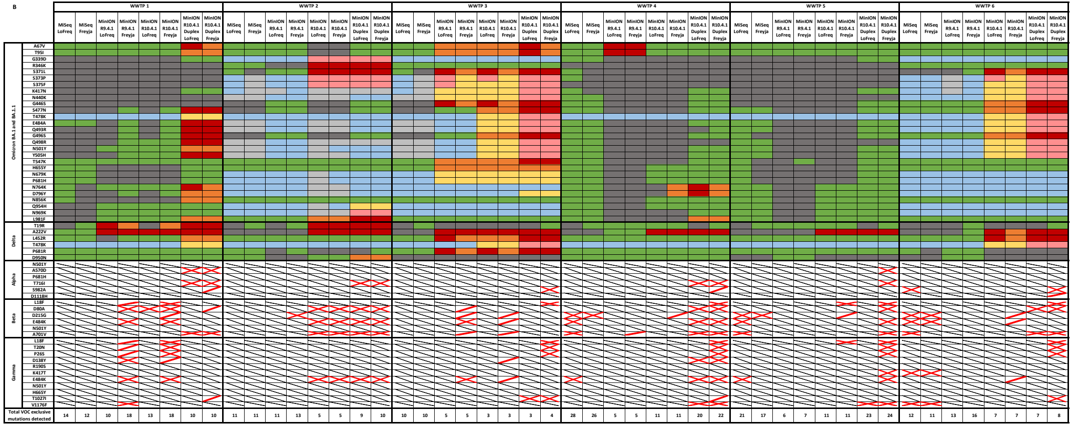

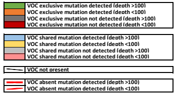

Figure S1: Overview of the signature mutations detected within each RNA mixture (A) and wastewater sample (B).

Table S1: Standard curves, limit of detection (LOD) and limit of quantification (LOQ) for the four targets of the duplex RT-qPCR allelic discrimination assays.

		Slope	Intercept	Efficiency	R^2	LOD	LOQ
**S:Del157/158**	Probe-Delta (FAM)	-3.59	42.51	89.75	0.99	7.65E+03	1.94E+04
	Probe-NoDelta (HEX)	-3.61	40.94	89.24	0.99	4.53E+03	1.24E+04
**S:Ins214**	Probe-BA.1 (FAM)	-3.45	39.45	95.00	0.99	1.93E+04	2.64E+04
	Probe-NoBA.1 (HEX)	-3.42	39.27	96.00	0.99	1.78E+04	3.55E+04
